# A ruptured uterus in a pregnant woman not in labor

**DOI:** 10.4314/pamj.v8i1.71048

**Published:** 2011-01-19

**Authors:** Damien Punguyire, Kenneth Victor Iserson

**Affiliations:** 1 Kintampo Municipal Hospital, Kintampo, Ghana; 2 University of Arizona, USA

**Keywords:** Pregnancy, grand multiparity, ruptured uterus, ultrasound diagnosis, nurse education, Ghana

## Abstract

Reducing maternal mortality constitutes one of the eight Millennium Development Goals. While significant progress has been made, system issues and professional training continue to affect maternal survival, especially when unusual, but deadly, complications arise. This rare case of survival after the rupture of an unscarred uterus in a grand multiparous woman from a remote village in Ghana illustrates how systemic transportation issues and limited access to advanced medical care put women with obstetric complications at risk. The usual clinical presentation of ruptured uteri and methods to prevent this catastrophic event are discussed. This case illustrates the systemic transportation issue that often limits access to prenatal and emergency care throughout much of the developing world and demonstrates how advanced training for emergency nurses and the use of ultrasound diagnosis can expedite difficult diagnoses and lead to maternal survival, even in the most adverse circumstances.

## Background

Reducing maternal mortality constitutes one of the eight Millennium Development Goals. While significant progress has been made, with a decrease from 422 maternal deaths/100,000 live births in 1980 to 251 maternal deaths/100,000 live births in 2008, system issues and professional training continue to affect maternal survival, especially when unusual, but deadly, complications arise [1].

This case illustrates the systemic transportation issues that often limit access to prenatal and emergency care throughout much of the developing world. It also demonstrates how advanced emergency nurse training and ultrasound capability can provide astute clinicians with the margin of time needed to save lives.

## Patient and case report

About 1 am, a 34-year-old G8 P7 woman from a remote village in Ghana experienced a sudden onset of abdominal pains, nausea, and lightheadedness. When it didn’t resolve by morning, she and her relatives began walking to the nearest road to get to the District Hospital. She had received no prenatal care and had vaginally delivered her previous seven children at home without complications with the help of a traditional birth attendant. All were alive and healthy; although she did not know her oldest child’s exact age, that woman now had three children of her own. The patient had never had abdominal surgery and was otherwise healthy.

Since her symptoms resolved somewhat while walking, the group stopped along the way to bathe, wash their clothes and eat. However, upon arriving at the small town, she experienced severe nausea, increased abdominal pain, and near-syncope. Her memory of subsequent events was hazy. A taxi, which her companions had to pay for in advance (she had only the government insurance provided to pregnant women, rather than the 15 Ghana Cedis ($10.70 U.S.)/year family health insurance), took her on the two-hour ride to the Kintampo Municipal (District) Hospital. She arrived at 7:10 pm.

**Figure 1:**
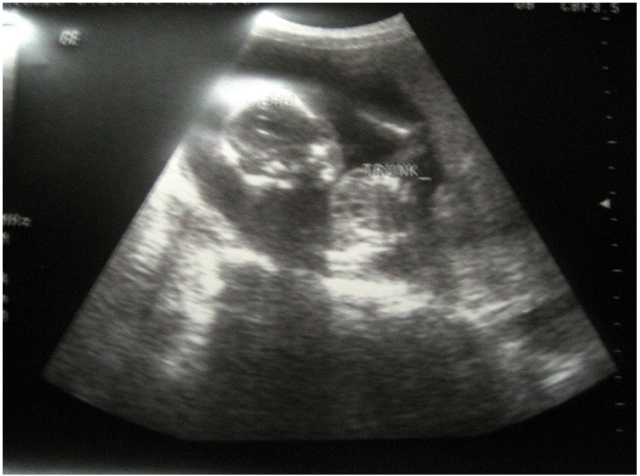
Ultrasound abdomen with live 17-week intrauterine fetus and large amount of free intraperitoneal fluid

Upon arrival, an emergency department nurse found that she had a distended, diffusely tender abdomen. (The nurse had advanced emergency medicine training through Columbia University’s sidHARTe Program: www.sidHARTe.org). Her vital signs were: BP, 70/40 mm/Hg; RR, 34/min; HR, 96/min; T, 34.8°C (axillary). The nurse immediately called the local surgeon who found a very pale woman with guarding and rebound tenderness. Suspecting an ectopic pregnancy, he first did an abdominal tap, which did not yield any blood. He immediately performed an abdominal ultrasound and visualized a 17-week intrauterine fetus with a heartbeat and a large amount of intraperitoneal free fluid (Figures 1). An ultrasound-guided abdominal tap yielded non-clotting blood. Laboratory studies that had been drawn on arrival showed Hgb 7.4 g/dL; Hct 27%; WBC 11.2 X 10^3^/µL . She was prepared for emergency surgery and entered the operating room at 8:20 pm.

Just before anesthesia induction, the patient suddenly said that she would not consent to the operation unless she could be assured that the pregnancy would be terminated. She said that she had enough children. Since the surgical staff couldn’t assure her of that, she refused anesthesia until she was informed (with no hyperbole) that, without the operation, she would quickly die. She immediately agreed to the surgery.

A transverse C-section incision was made. Immediately, a tiny umbilical cord appeared, floating in the blood-filled abdomen. The fetal head was then seen protruding from a macerated uterine fundus with the placenta attached in the right uterine cornu. (This was a "cornual pregnancy," gestation in the horn of the uterus).

The now dead fetus and remaining placental tissue were quickly removed from the uterus through the gaping fundus. The fundus was then clamped and oversewn. At that point, the patient was hypotensive (80/40 mmHg) and receiving both packed erythrocytes and crystalloid. About 4L of sanguineous fluid was removed from her abdomen. After ascertaining that bleeding had ceased, the fallopian tube that had not been removed with the uterine fundus was ligated and bisected.

Following surgery, the patient’s condition improved with fluids and blood administration. The next day she was stable, with normal vital signs, a postoperative ileus, and pain only at the incision site. She was discharged two days later.

## Discussion

**Incidence**

Uterine rupture without prior C-section is a rare and catastrophic event, often resulting in the death of the baby, extensive damage to the uterus, or maternal exsanguination.[2] Rupture of the unscarred uterus occurs in only 0.006% (<1 in 10,000) of pregnancies in the most-developed countries [3].

Uterine rupture occurs more commonly in pregnant women with prior C-sections. They suffer uterine ruptures <1% of the time in the most-developed countries, between 0.1% (Israel) and 19% (Nigeria) in the less-developed countries, and between 0.2% (Madagascar) and 25% (Ethiopia) in the least-developed countries [1].

Yet, in regions without autopsies for sudden deaths, the actual incidence is questionable. The number of cases and deaths, particularly those that are not evaluated or that occur in health facilities, likely are underreported.

**Death**

In less-and least-developed countries, uterine rupture is a not-uncommon cause of maternal mortality [2]. In South Africa between 1999 and 2001, for example, ruptured uteri caused 6.2% of deaths due to direct causes and 3.7% of all deaths (1.9% due to rupture of unscarred uteri and 1.8% due to rupture of scarred uteri) [4]. In one Indian study, it caused 9.3% of maternal deaths [2].

**Risk Factors**

The pregnant uterus rarely ruptures without external trauma when not in active labor, but cornual pregnancy and multiparity, particularly grand multiparity (i.e., seven or more children), [5] are two reported risk factors [6].

Ruptures during labor, while uncommon, usually occur in women with prior C-sections or abdominal surgery. A major contributor is obstructed labor, and Black African women are especially at risk, having a high incidence of contracted pelvis [7].

**Common Presentation**

Schrinsky and Benson’s description of the normal course of uterine rupture suggests why complications may still be so common and illustrates why ultrasound is vital to early diagnoses: “The catastrophic event, paradoxically, is often somewhat insidious in its onset and development, and the textbook picture of absent fetal heart tones, sudden severe, shearing abdominal pain, cessation of uterine contractions, bleeding and shock is quite often simply not present. Because of this, the diagnosis is often not considered, or, perhaps, considered briefly and disregarded because of its relative rarity; this is especially true if the accident occurs before the onset of labor, in an inconspicuous clinical setting” [6].

**This Case**

Throughout Africa, patients live in remote sites with limited transportation, which can delay and limit their access to medical care. Our patient probably survived due to an intact serosa maintaining intrauterine integrity for about 18 hours after the initial tear, since the preoperative ultrasound showed that the fetus still had a heartbeat. Contributing to her survival was her family’s ability to pay for transportation to (barely) reach the hospital alive. Also vital to her survival were ED nurses with specialized training, who immediately recognized that she was critically ill and initiated the surgical response. (This model for providing advanced emergency medical training to ED nurses in the least-developed countries is now spreading to other countries)

Finally, providing the patient with accurate prognostic information at a critical point—as she was about to be anesthetized (appropriately saying “you are going to die”)—helped her make the decision to proceed with the intervention, for which she was subsequently very grateful.

**Prevention**

WHO states that reducing the incidence of unscarred uterine ruptures requires reducing unwanted pregnancies, particularly for women of high parity; increasing the ability to access obstetric services including C-section for obstructed labor; using innovative methods, such as symphysiotomy [8] or C-section with local anesthesia where conventional C-section facilities are not available; and using guidelines to ensure that only safe doses of misoprostol are used for labor induction.

## Conclusion

Pregnancies in grand multiparous women occur commonly in Africa, as in many other areas of the world. In these same areas, transportation problems often make accessing obstetric care difficult. Despite these issues, as this case demonstrates, advanced training for emergency nurses, general physicians with surgical skills, and the use of ultrasound diagnosis can expedite difficult diagnoses and lead to maternal survival.

## Competing interests

There are no financial or competing interests associated with this manuscript.

## Authors’ contributions

Both authors participated in diagnosing and treating the patient in this case, drafting this article, and approving the final version for publication.
